# Stuck in the blend: Challenges faced by students enrolled in blended programs of Masters in Health Professions Education

**DOI:** 10.12669/pjms.35.4.12

**Published:** 2019

**Authors:** Noor-i-Kiran Naeem, Rehan Ahmed Khan

**Affiliations:** 1 Dr. Noor-i-Kiran Naeem, FCPS, MSc. Med. Department of Medical Education, Aziz Fatimah Medical and Dental College, Faisalabad, Pakistan; 2 Dr. Rehan Ahmed Khan, FCPS, FRCS, JM-HPE, MSc. HPE. Islamic International Medical College, Riphah International University, Islamabad, Pakistan

**Keywords:** Blended learning, Challenges, Health Professions Education, Hybrid learning, Problems

## Abstract

**Objectives:**

The advent of computer technology and widespread use of internet has given rise to e-learning and blended programs all over the world. The aim of this study was to explore problems faced by students enrolled in blended program of MHPE in Pakistan.

**Methods:**

This was a qualitative exploratory study done between October 2017 and February 2018. Data included semi-structured individual interviews of eighteen students and four facilitators involved in blended MHPE programs of three leading Universities of Pakistan. Nine hundred and two students’ reflective essays were also included for data triangulation. Data was organized in Atlas-ti and analyzed through thematic analysis using Revised Community of Inquiry framework.

**Results:**

Seventy open codes were condensed to fifteen sub-themes and five themes. Learner related problems comprised difficulty in self-regulation and self-directed learning as emphasized by the facilitators whereas students quoted teacher related problems focusing on feedback provision. Cognitive issues included huge cognitive load with engagement issues. Students also highlighted issues with social interaction encompassed difficulties in interacting with facilitators and managing group dynamics. Both students and facilitators agreed on institutional issues focused on limited resource provision, unsatisfactory administrative support and financial issues.

**Conclusion:**

Students of MHPE are challenged with variety of issues in blended learning program relating to self-regulation, heavy cognitive load with engagement, social interaction especially with facilitators and managing group dynamics Addressing these issues can improve the experience of these students in blended programs.

## INTRODUCTION

Blended programs of Masters of Health Professions Education (MHPE) in Pakistan have increased from one in 2009 to eight in 2018.^1^ These programs are primarily designed on rotation model, including traditional component of face to face (f2f) sessions, alternating with distance learning phase of two to three months.^2^

Blended programs offer many advantages. Firstly, they provide an opportunity to students from diverse specialties and busy routine to continue their studies. Secondly, apart from providing pedagogical richness, there is varying degree of social interaction, flexibility, and ease of revision for the students in a cost-effective approach.^3^ Moreover, blended learning provides teachers flexibility in addressing educational standards and maintaining curriculum authenticity while integrating digital content and learning experiences to engage 21^st^ century learners.^4^

However, not all the ‘good’ is without its ‘bad’. Blended programs may fall into a trap of issues that the students face during learning activities. It remains debatable whether there is true blend in learning process or just in delivery mode and distance.^5^ It remains hard to keep a check on how to keep students motivated towards their learning. Moreover, distance learning phase of such programs demonstrate lack of socialization but also inability to accommodate struggling students.^6^

The MHPE Programs of Pakistan are at stage of early implementation with limited evaluation data available on available educational policies across the country.^7^ Adopting a constructivist-collaborative approach, these MHPE programs demand a sophisticated blend between the needs of all stakeholders (students, facilitators and institutions) in order to maintain interactivity and dynamicity they offer. Considering the diverse nature of students enrolling with traditional styles of learning, it is high time to explore the issues faced by them. The purpose of this study was to explore the students’ and facilitators’ perspective about problems faced by students in blended MHPE programs.

## METHODS

This qualitative exploratory study was done from October 2017 to February 2018. Eighteen students and four facilitators were included from three universities from three different cities of Pakistan.

### Data Collection

Participants underwent individual, semi-structured audio-recorded, telephonic interviews. Two sets of six open-ended questions, each for students and facilitators, were designed on the basis of Revised Garrison Community of Inquiry framework, which has been developed as a means to investigate effective online and blended learning environments in higher education.^8^ After pilot testing, the participants were approached, and informed consent was taken. The interviews were transcribed in English language and sent back to participants for member checking. Data saturation started occurring at the fourteenth student interview. However, four more interviews were recorded to countercheck the recurrence of codes in data.

Data source triangulation was achieved by obtaining 902 students’ reflective essays from one university’s archival record after approval in order to check the consistency of findings.

### Data Analysis

The interviews and essays were organized in Atlas-ti software for analysis. We adopted the Framework method of analysis with priori coding, using the Revised Garrison Community of Inquiry framework.^9^

## RESULTS

**Fig. 1:**
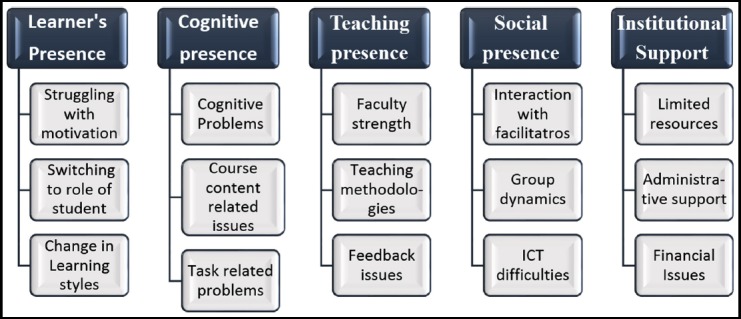
Overarching themes and subthemes in the study taking Community of Inquiry as Conceptual framework.

Seventy open codes were condensed to fifteen subthemes and five main themes. Fig.1 shows hierarchy of themes and subthemes identified.

### Problems related to learner

Motivations behind joining the course was discussed a major factor relating to learner’s difficulty with self-regulation and self-directed learning. Fourteen students joined the program because of being ‘in trend’. Facilitator 1: *“Entering these programs has become a band wagon phenomenon…. everyone is not equally motivated.”* Other reasons quoted by students included career up gradation, personal interest, feasibility as well as being recommended by others. Students also found it challenging to become a student again, and demonstrated habits of procrastination due to poor time management. Facilitator 2: *“They are all rabbits. They say that we already know a lot and they can take slumber… later when they realize this time is almost up, they do assignments hurriedly.”* Challenge of shifting from passive learning to self-directed learning was discussed by all four facilitators but only three students. *Student 17 quoted: “We are just very much used to the pampering from teachers and getting everything ready on the plate*.”

### Problems related to Cognition

Heavy cognitive load with lack of hands-on practice in workplaces was highlighted by most students. *Student 17: “If I had some first-hand experience before, then I would have known the exact issues regarding teaching and curriculum designs*. “Students felt frustrated at times because of completing difficult tasks given during class session. *Student 5: “I was feeling stuck while reading it because I still could not develop the exact skill of skimming an article*. “Facilitators emphasized on issues of keeping students engaged during classroom activities. Facilitator 4: *“Problem is that there will always be so many distractions. Facebook will always be open and there will be WhatsApp….”*

### Problems related with Social Interaction

Reasons for struggling in managing group dynamics included being passive in group, not being able to work in a new group, allocating tasks to team members and dealing with seniors in group. *Student 5: “Challenging part of working in a group was, keeping everyone along and completing your task in a given time-frame”*. Diversity in students’ backgrounds and academic levels also caused issues in working together. Issue of bullying by other students was also discussed. *Student 18: “I used to feel the taunts and bullying by them …. They made a point to make other feel low of himself.”* Students were hesitant to interact with the facilitators during the distance learning phase. *Student 2: “At home, it was very difficult to contact our facilitators. Sometimes, they read the email immediately but sometimes couldn’t reply on time.”*

Twelve students and two facilitators pointed at the paperless setting being a burden instead of facilitating learning. *Student 7: “…. a paperless environment was austere… to think about a new field and then using computer with less than optimal skills to reproduce was difficult.”* Discussing the use of learning management site(LMS), students felt frustrated at the server not working properly. *Student 18: “…it was the last day to submit thesis but it was not being submitted due to server issues.”* Also, LMS appeared to be boring probably because of being used scarcely. Facilitator 4: *“Moodle is just used for putting academic stuff; we are not using it to its true potential.”*

### Problems related to Facilitators

Both groups pointed on a low facilitator: student ratio as well as lack of local faculty. Facilitator 4: *“We do not have very good ratio of faculty to students, and mostly faculty is visiting.”* They also pointed out those boring and non-engaging teaching strategies hindered student’s learning.” Varying teaching styles with conflicting views caused confusion among the students. *Student 5: “I felt every teacher had his own way of teaching same thing in a different way. That was difficult to grasp.”* All students agreed on lack of timely and clear feedback in distance-learning phase. *Student 8: “Now if you have shortage of time, feedback got delayed, all of your schedule is effected and there is a lot of mental stress.”* Students agreed that absence of feedback made them at a loss about their progress. *Student 5: “…. how can I be confident that I have learnt up to the mark without a feedback regarding my assignments?”*

### Problems related to Institutional Support

Twelve students mentioned lack of proper classroom environment. *Student 5: “It was a cramped place with no ventilation…”* Four students discussed issue of inaccessible library for usage. *Student 9: “Library access was limited after working hours. I really wanted that*. “Both groups reported lack of proper communication with the administration. *Student 5: “I am facing lazy behavior from the University in providing the transcript and the degree*. “Five students discussed strict, inflexible university rules causing anxiety. *Student 12: “University had kept very strict rules which the students couldn’t keep up with and they left. Hence this could be counted as program failure.”* One facilitator pointed out lack of scholarship for students. Facilitator 2: *“I know many people who were so bright, but they couldn’t manage financially to get into the program. Unfortunately, institutions do not provide any assistance in this context*.

## DISCUSSION

Our study highlighted many issues in learner’s presence linked with motivation and self-regulation. Ahsan et al asserted that by knowing about participants’ motivations to learn can eventually influence their qualification’s impact.^1^ Being aware about one’s interests and potential seems crucial. Hence, following others like a band wagon phenomenon, without probing one’s interest in it, are closely linked factors that can determine future performance of the student.^10^ Chang et al discusses role of bandwagon phenomenon in terms of Johari’s window as a part of being aware of oneself. Those who followed the band-wagon phenomenon fell into the blind window where not they themselves or others knew about their potential. 11

Valerica emphasized on having right kind of mindset towards learning to have higher academic performance as compared to those making less efforts in changing their attitudes with time.^12^ Hence being flexible in accepting new learning methodology and in becoming a student again becomes a part and parcel of the blended program which was a challenge for the students in our study.

There were students who found problems in developing autonomy and becoming self-directed learners. Jones J claimed that the ‘most successful learning takes place outside the classroom’. In order to accomplish this task students must be taught the positive attributes of ‘how to learn’ by themselves’.^13^ The attributes related to self-regulation include time management skills, study environment management, learning management, prioritization as well as keeping intrinsic motivation to complete a task given to the students.^14^

**Fig. 2:**
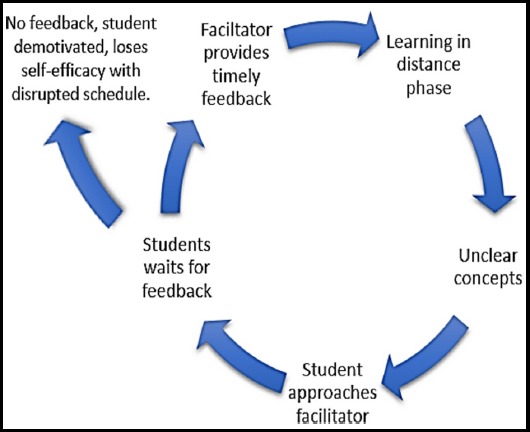
Feedback loop identified in the study.

Much blame was given to the previous traditional teacher-led spoon fed learning approach which was difficult to adapt. This transition of students from being passive learner to active learners may be challenging but it can be overcome by providing good mentoring and support from the teachers.^14^ Also, it is up to the facilitator in such programs to make sure that students participate actively in the classroom activities. Moreover, there were instances when the students felt lack of support from the teachers in terms of their availability and timely feedback provision. Getting a timely feedback from the facilitators not only encourages the students to work harder and promote their engagement in learning but also clarify their learning needs and deficiencies as shown in feedback loop identified in the study (Fig.2).^15^,^16^

The cognitive presence in our study had problems in both the inquiry process as well as in collaboration with others. Physical fatigue during the contact sessions accounted for many of the issues that these students raised including the long working hours which caused students difficulty in exploration, construction, resolution, and understanding through collaboration and inquiry. Also along with innate cognitive difficulty, there were issues with group dynamics amongst the students. There was also lack of engagement in class activities which led to lack of interest and practice.

Content organization plays a key role in the smooth experience in any educational program. Chandler and Sweller debated that the degree of interactivity between instructional elements and course content determines intrinsic cognitive load.^17^ This goes hand in hand with the fact that blended learning inducts several types of instructional methodologies including ICT, which adds on to the intrinsic load related to the content of the course itself. Hence instead of facilitating learning, these technologies may at times impede the learning process of the student itself.^18^ Misusing social media, for instance had been reported to become a hindrance instead of facilitating learning. Whereas the interaction between student and facilitator is crucial through such media, keeping a boundary in such media is also important.^19^

Although blended learning is based on constructivist-collaborative approach, students faced issues of mismanaged group interaction. In contrast, Ahmed emphasized on group dynamics amongst students to be smooth to ensure task completion and learning advancement.^20^

Moskal et al. identified three main goals of institutions using blended learning programs that such programs should be able to enhance pedagogy, increase flexibility and access for students as well as provide a cost-effective resource usage.^21^ In contrast, our study demonstrated a varied response with lack of support regarding infrastructure regarding providing classroom environment in terms of ambience, furniture and internet provision.

### Limitations of the study

The number of facilitators was few in our study. Although they were involved in the study to explore the problems faced by students, the challenges faced by them and institutions still need to be explored.

## CONCLUSION

Blended learning is an upcoming modality all over the world due to the feasibility and flexibility it offers. However, with this autonomy, student’s responsibility also increases with demanding self-regulation and self-directed learners. Proper support, timely feedback and easy availability of the facilitators can facilitate students in adopting to challenging shift of traditional learning to blended learning. The Facilitators play a myriad of roles in blended programs, from instructor to mentor and by providing timely feedback, and being approachable, they can facilitate quality learning. Institutional support can guarantee a smooth educational experience for these students. The study suggests that regular evaluation of blended programs should be in practice to identify needs of the stakeholders including students. By answering to the students’ needs and problems, improvements in students’ experience can be ensured.
